# Effect of adiponectin level and genetic variation of its receptors on diabetic retinopathy: A case–control study

**DOI:** 10.1097/MD.0000000000014878

**Published:** 2019-03-15

**Authors:** Wen-Ling Liao, Yung-Hsiang Chen, Ching-Chu Chen, Yu-Chuen Huang, Hui-Ju Lin, Yng-Tay Chen, Bo Ban, Chia-Ming Wu, Ya-Wen Chang, Ai-Ru Hsieh, Fuu-Jen Tsai

**Affiliations:** a Graduate Institute of Integrated Medicine, China Medical University; b Center for Personalized Medicine, China Medical University Hospital; c Chinese Medicine Research Center, Research Center for Chinese Medicine & Acupuncture, China Medical University; d Department of Medical Research, China Medical University Hospital; e Department of Psychology, College of Medical and Health Science, Asia University; f School of Chinese Medicine, China Medical University; g Division of Endocrinology and Metabolism, Department of Medicine, China Medical University Hospital; h Human Genetic Center, Department of Medical Research, China Medical University Hospital, China Medical University; i Department of Ophthalmology, China Medical University Hospital; j Graduate Institute of Food Safety, National Chung Hsing University, Taichung, Taiwan; k Department of Endocrinology, Affiliated Hospital of Jining Medical University; l Chinese Research Center for Behavior Medicine in Growth and Development, Jining, Shandong, China; m Graduate Institute of Biostatistics, Department of Public Health, China Medical University; n Division of Pediatrics Genetics, China Medical University Children's Hospital, Taichung, Taiwan.

**Keywords:** CDH13, diabetic retinopathy, genetic variants, plasma adiponectin

## Abstract

Adiponectin (APN) and its receptors have been reported to be associated with metabolic phenotypes. To better understand the effects of APN levels and its receptors on diabetic retinopathy (DR), we investigated the association of the plasma APN level and variations in APN-related genes with DR, individually and in combination.

Patients with type 2 diabetes (T2D; N = 1604), above 20 years of age from the Taiwanese population participated in the study. Demographic information, blood pressure, and serological markers were recorded at enrollment. Genomic DNA was isolated and genotyped. The plasma APN levels were measured by enzyme-linked immunosorbent assay.

T2D patients with DR (N = 632) had diabetes for a longer duration, and had higher HbA1c, and systolic and diastolic blood pressure compared to those without DR (N = 972) (*P* < .001, for all the parameters). Overall, 10 single nucleotide polymorphisms (SNPs) in *ADIPOQ* and *CDH13* susceptibility loci were associated with DR. Gene risk score (GRS) was calculated based on 10 SNPs for each subject and the cumulative effect of genes was observed. Among the subjects with plasma APN level (N = 518), natural logarithm (LN) of APN (LN [APN]; odds ratio [OR] = 1.63, 95% confidence interval [CI] = 1.19–2.25) and GRS (OR = 1.90, 95% CI = 1.11–3.26 for middle range of GRS, and OR = 2.61, 95% CI = 1.48–4.59 for high range of GRS) were independent risk factors for DR after adjustment for other parameters.

In conclusion, the plasma APN level and the genetic variations in adiponectin receptors were associated with DR.

## Abstract 2

Supplemental Digital Content is available in the text

## 1 Introduction

Diabetic retinopathy (DR) is one of the major microvascular complications in diabetes patients. Several risk factors are identified to be related to DR, including long duration of diabetes, elevated glycosylated hemoglobin levels, dyslipidemia, high blood pressure, and chronic hyperglycemia. For the development and progression of DR, hyperglycemia plays a central part in a cascade of damaging effects mediated by cytokines and growth factors that produce oxidative stress, abnormal glycosylation, lipid peroxidation, and subsequent generation of inflammatory elements. In addition to metabolic and physiological factors, genetic factors also influence the pathogenesis of DR.^[1,2]^ The risk of DR is about 2- to 3-fold higher in siblings of affected individuals compared with those in the siblings of diabetic patients without DR.^[3,4]^

Adiponectin (APN), a 30-kDa adipocyte-derived vasoactive peptide is the most abundant circulating hormone secreted by the adipocytes. APN is an important contributor to peroxisome proliferator-activated receptor-γ (PPARγ)-mediated improvements in insulin sensitivity.^[5]^ APN also has anti-inflammatory and anti-atherosclerotic effects on endothelial cells, which are mediated by a decrease in vascular inflammation, formation of foam cells, and cell adhesion, all of which are involved in the initiation and progression of vascular lesions. A number of studies have reported the relationship between the plasma APN level and a number of metabolic conditions, including obesity, insulin resistance, type 2 diabetes (T2D), and cardiovascular diseases.^[6–14]^ However, the association between the levels of circulating APN and the DR status has not been found to be consistent.^[12,14]^

The APN cellular signaling is mediated by 3 major receptors, namely AdipoR1, AdipoR2, and T-cadherin. AdipoR1 is involved in the activation of 5′ adenosine monophosphate-activated protein kinase, and AdipoR2 is involved in PPAR-α pathways, leading to increased insulin sensitivity.^[15]^ The third candidate receptor, T-cadherin, encoded by *CDH13*, has been implicated in the modulation of angiogenic activities in cultured endothelial cells^[16–19]^ but the results have been inconsistent. Genome-wide association studies have identified links between CDH13 and cancer,^[20]^ metabolic syndrome, metabolic phenotypes,^[21]^ T2D,^[22]^ and ischemic stroke.^[23]^

Taken together, these results indicate that APN and APN receptors could play roles in the development of DR. However, limited number of studies has focus on the combined effect of APN and APN receptors on DR. In the present study, we investigated the association between the APN level and the variance of APN-related genes on the DR status, individually and in combination.

## 2 Material and methods

### 2.1 Study population

A total of 1604 T2D patients, aged 20 years and older were included in this study, including 632 subjects with retinopathy (DR, case) and 972 subjects did not have retinopathy (non-DR, control) at the time of enrollment. All of the subjects were recruited from the China Medical University Hospital (CMUH), Taichung, Taiwan. Diabetes was diagnosed based on medical records and fasting plasma glucose levels according to the American Diabetes Association Criteria.^[24]^ Patients with type 1 diabetes, gestational diabetes, and maturity-onset diabetes of the young were excluded from this study. The participants were of Han Chinese ethnicity characteristic for 98% of the population in Taiwan. All T2D patients underwent complete ophthalmologic testing, including corrected visual acuity, fundoscopic examination, and fundus photography. An expert ophthalmologist graded DR according to the International Clinical DR Disease Severity Scale proposed by the American Academy of Ophthalmology.^[2,25]^ The study was approved by the CMUH Institutional Review Board (CMUH103-REC2-071) and informed consent was obtained from all participants. The study was conducted in accordance with the tenets of the Declaration of Helsinki.

### 2.2 Plasma APN level measurements

The plasma APN measurement was assessed by enzyme-linked immunosorbent assay (ELISA) kit (Invitrogen, MA). Plasma samples or standards were added to each microtiter plate wells with a biotin-conjugated antibody that specific to protein candidates. The reactive time was performed according to the manufacturer's instructions. Finally, the optical density (OD) was measured at 405 nm. The concentration of protein candidates in the samples was calculated by fitting the OD value of the samples into the standard curve.

### 2.3 DNA extraction

Blood was collected in a tube with K3 EDTA to extract deoxyribonucleic acid (DNA). Genomic DNA was extracted from peripheral blood leukocytes by using the Genomic DNA kit (Qiagen, CA). DNA quality was assessed by visual inspection after running 1.2% agarose gels and by calculating absorbance ratio OD260 nm/280 nm. DNA quantification was measured using Picogreen dsDNA reagent. Degraded samples or those with low DNA concentration were excluded from following experiments.

### 2.4 Genotyping and imputation

We performed genotyping or imputation for all the single nucleotide polymorphisms (SNPs) in 4 susceptibility loci, *ADIPOQ, ADIPOR1, ADIPOR2,* and *CDH13* using Illumina HumanHap550-Duo BeadChips or Affymetrix-TWB chips. Genotypic data were quality-controlled, and SNPs were excluded from further analysis if:

(1) Minor allele frequency was less than 5% in non-DR T2D controls,
(2) the total call rate was less than 95% for both DR and control patients, or
(3) significantly departed from Hardy–Weinberg equilibrium (HWE) proportions for controls (*P* < .05).

**Figure 1:**
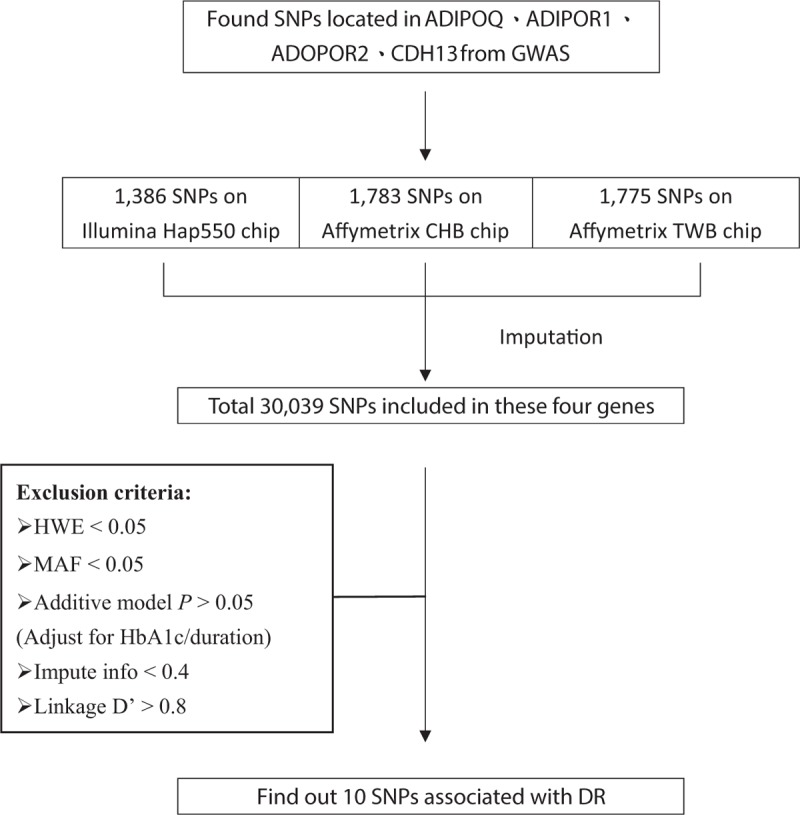
The flow chart for selecting genetic markers into genetic risk score.

For the untyped SNPs, genotype imputation was performed according to the methodology of Howie et al^[26]^ implemented in impute v2 (http://mathgen.stats.ox.ac.uk/impute/impute_v2.html). The panel from 1000 Genomes Project was used as reference for imputation, and the software chose the best-customized reference set for each individual. The deviation from the additive model was tested for SNPs and those with *P* value > .05 were excluded. More, SNPs with low imputation quality (info <0.4) and those in the same gene showing strong disequilibrium with each other (D’ >0.8) were excluded from further analysis. The flow chart for genetic selection was presented in Figure 1. And the pair-wise linkage disequilibrium between the 9 SNPs on CDH13 loci was also presented in Figure S1.

### 2.5 Selection of genetic markers

After applying all the selection criteria mentioned above, we selected a total of 10 SNPs as genetic markers by using a multivariable logistic regression analysis adjusting for the T2D duration and HbA1c level. The genotypes were coded in the additive model as “0” for non-risk allele homozygotes, “1” for heterozygotes, and “2” for risk allele homozygotes for the 10 SNPs. The weighted genetic risk score (GRS) for each individual was calculated based on the number of risk alleles weighted by the effect size (logarithm of odds ratios [ORs]), according to the following equation:^[27]^ weighted GRS = 10/2.407 × ((rs16861205_risk × 0.310) + (rs16958347_risk × 0.147) + (rs112016995_risk × 0.343) + (rs4315313_risk × 0.183) + (rs16959303_risk × 0.177) + (rs6565142_risk × 0.177) + (rs1862830_risk × 0.149) + (rs1862832_risk × 0.180) + (rs79769659_risk × 0.555) + (rs8053692_risk × 0.186)). The weighted GRSs were divided into 3 equal groups to calculate the cumulative effect.

### 2.6 Statistical methods

The values were calculated as means ± SD, median with range, or percentages. The mean/median levels of continuous variables in the different groups were compared using 2-independent *t* test (for comparison of 2 groups), 1-way analysis of variance (for comparison of 3 or more groups) or related nonparametric methods (Mann–Whitney *U* test or Kruskal–Wallis test). The percentage of categorical variables in the different groups of T2D patients was compared using the *χ*^2^ test or the Fisher exact test. The observed genotype frequencies for all the polymorphisms in the controls were examined to determine their deviation from the HWE using a goodness-of-fit *χ*^2^ test with 1 degree of freedom. The distribution of genotype (in dominant, recessive, and additive models), and allelic and haplotype frequency in the different groups of T2D patients were analyzed using the *χ*^2^ test or the Fisher exact test for differences in proportions. The APN values were log transformed to induce normality of distribution. The association of plasma APN and GRS with DR was analyzed using multiple logistic regression analysis. The potential confounders were selected into models by the backward method. All the models were adjusted for the following confounders, including age, duration of diabetes, body mass index (BMI), HbA1c, and systolic blood pressure (SBP). The interaction term between the plasma APN level and GRS was investigated. The statistical analyses were conducted using the SPSS software, v12.0 for Windows (IBM, Armonk, NY), and *P* value less than .05 (2-sided) was considered to be statistically significant.

## 3 Results

### 3.1 Characteristics of the study participants

Among the 1604 T2D patients included in this study, 632 had DR (case) and 972 did not have DR (non-DR, control). No statistically significant differences in gender, age distribution, and BMI were observed between the 2 groups. However, the duration of diabetes affliction, HbA1c level, SBP and diastolic blood pressure in the DR group were significantly higher compared to that in the control (*P* < .001 for all the parameters; Table 2). Among the subjects for whom clinical information was available, the DR cases had higher values for fasting glucose and albumin to creatinine ratio and lower values for low-density lipoprotein and estimated glomerular filtration ratecompared to the non-DR subjects (Table 2).

**Table 2:**
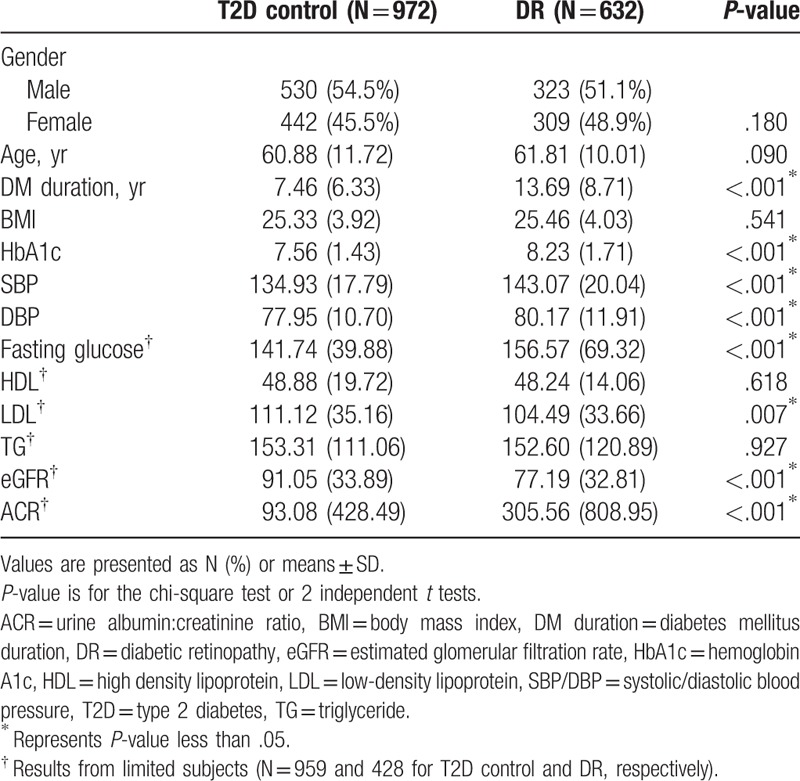
Demographics of the overall population.

### 3.2 Associations between genetic polymorphisms of ADIPOQ, ADIPOR1, ADIPOR2, and CDH13 and DR

We determined the association between the genetic polymorphisms of *ADIPOQ*, *ADIPOR1*, *ADIPOR2*, and *CDH13* and DR in the study population. A total of 10 SNPs, including 1 SNP from the *ADIPOQ* loci and 9 SNPs from the *CDH13* loci were selected as genetic markers using a multivariable logistic regression analysis adjusting for the duration of diabetes and HbA1c level (Table 1).

**Table 1:**
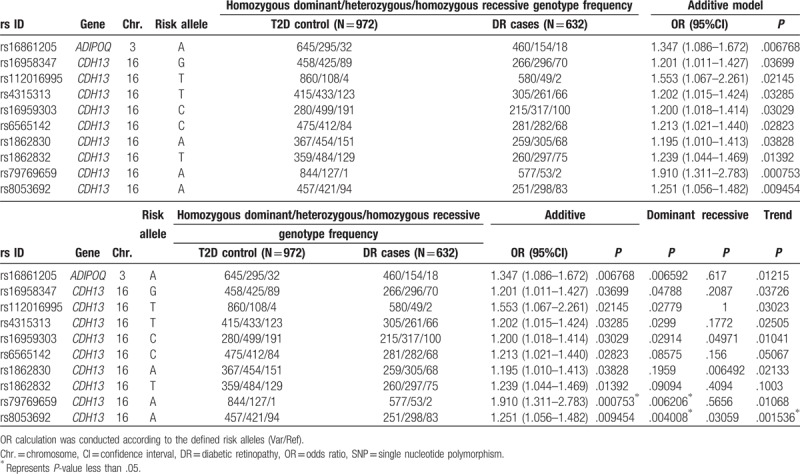
Association between the 10 single nucleotide polymorphisms included for determining the genetic risk score and diabetic retinopathy status in the Taiwanese population.

Furthermore, we calculated the multiplex GRS for each individual. The cumulative effect of the 10 selected SNPs from the logistic regression model was assessed by counting the number of risk genotypes in each individual, and the weighted GRS was calculated based on the logarithm OR of the susceptibility SNPs (the details regarding the equation of GRS are provided in Material and Methods). The mean number of risk alleles was 12.35 ± 2.02 (range 6–18), and the mean weighted GRS was 14.11 ± 1.81 (range 7.81–18.78). The distribution of the risk alleles and weighted GRSs is shown in Figure S2. All the patients were divided into 3 groups based on the number of risk alleles. The DR risk increased with the number of risk genotypes (*P* = 7.20 × 10^−11^; Cochran–Armitage Trend test), which suggests a cumulative effect of these 10 SNPs on the DR risk.

### 3.3 APN level is different among different stages of DR patients

**Figure 2:**
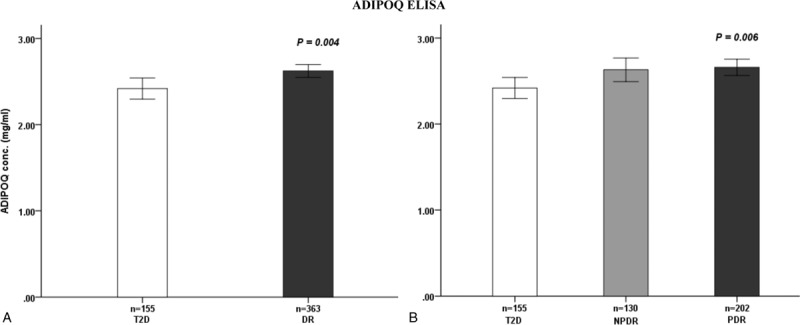
Plasma adiponectin level in diabetic patients. A, Comparison of the levels in diabetic subjects without (T2D) and with retinopathy (DR). B, Comparison of the levels in diabetic subjects without retinopathy (T2D), with NPDR), and with PDR. DR = diabetic retinopathy, NPDR = nonproliferal retinopathy, PDR = proliferal retinopathy, T2D = type 2 diabetes.

Among the total study population (1604 T2D patients), we measured the APN levels, using ELISA, in 518 plasma samples, from 155 non-DR subjects and 363 DR subjects. The mean values of the LN [APN] levels were significant different between T2D subjects, with and without retinopathy (non-DR vs DR; *P*-value = .004, for independent *t* test) and between non-DR subjects and PDR subjects (non-DR vs PDR; *P*-value = .011, for ANOVA test, with post-hoc Scheffe test) (Fig. 2).

### 3.4 Effect of the combination of plasma APN level and genetic factors on the DR status

In the multivariate logistic regression model, LN [APN] and GRS were independent risk factors for the risk of DR after adjustment for age, the duration of diabetes affliction, HbA1c, BMI, and SBP. The OR (95% confidence interval [CI]) was 1.65 (1.19–2.27) for plasma APN level. Compared with individuals in the lowest range of weighted GRS, the ORs (95% CI) for those in the middle and high range were 1.89 (1.10–3.25) and 2.56 (1.48–4.54), respectively (*P* = 1.86 × 10^−4^; Cochran–Armitage Trend test). However, no significant interaction term was observed between the plasma APN level and GRS (Table 3).

**Table 3:**
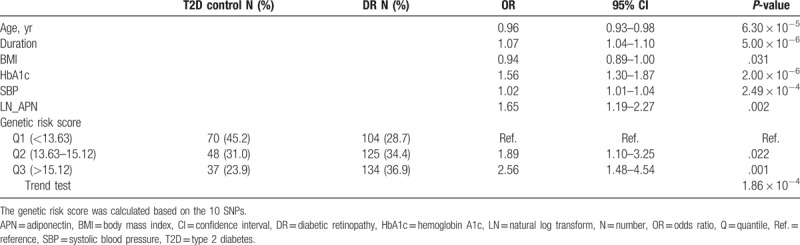
The effect of combination of plasma adiponectin level and genetic effects on DR status in multivariate logistic regression model.

## 4 Discussion

In the present study, we determined the effect of increased plasma APN level and GRS, which incorporated the variants of *ADPIOQ* and *CDH13*, on the DR status.

APN is a cytokine secreted by adipocytes and binds to its receptors to regulate lipid/glucose metabolism and anti-inflammatory effects.^[28]^ Previous research has shown that the level of APN is associated with the DR status in various ethnic groups.^[12,14]^ In the present study, the APN level was an independent risk factor for DR after adjusting for age, DM duration, HbA1c, and SBP, which are the potential confounding factors. Our result was consistent with that of meta-analysis done by Rodríguez et al, who showed that higher APN levels were associated with DR. They analyzed 3 controlled cross-sectional studies including 324 DR and 983 non-DR subjects from Korea and India.^[14]^ However, many studies report that a protective effect of APN against DR clinically and experimentally.^[12,29,30]^ The meta-analysis by Fan et al. found a significant negative association between the APN concentrations and severity of DR. Therein, the authors analyzed the results of 19 studies, including a total of 1545 cases and 1502 controls, predominantly belonging to Chinese Han dynasty.^[12]^ The possible reasons for these contradictory results include confounding factors (eg, obesity), measurement biases, and reverse causation. Studies have shown that APN levels are influenced by various factors, such as the degree of obesity, age, blood lipid, gender, smoking,^[31]^ glucose level, kidney function, the form of APN measured, genetic background, and anti-diabetic/cardiovascular drug used,^[32,33]^ such as thiazolidinediones or fenofibrates. Moreover, the increased levels of circulating APN observed in microvascular complications could result from a compensatory mechanism to counter inflammatory processes that occur as part of the pathophysiology of microvascular disease complication in the absence of macrovascular complications.^[14]^

Previous researches have demonstrated that heredity plays a key role in the pathogenesis of DR in various ethnic groups.^[1]^ In the present study, the cumulative effect of genetic variation of APN receptors on the DR status was observed. The effect of APN is mediated by 3 major receptors, namely AdipoR1, AdipoR2, and T-cadherin. When APN binds with the receptors, the cellular signaling proceeds and maintains the metabolic homeostasis of glucose and lipids, and might also affect the endothelial cell angiogenesis and increase the capillary permeability. Therefore, the variability in the receptors mediating the APN action could also play a role in DR. The effect of genetic polymorphism of *ADIPOQ* on DR was investigated recently.^[34,35]^ A significant association between *ADIPOQ* polymorphism and DR was observed in a study on 672 Indian patients (rs2241766 (T45G) *P* = .0007)^[35]^ but not in the study on 517 Chinese patients.^[34]^ In the present study, rs16861205 which was associated with DR was not linked with rs2241766 (D’ 0.184 in CHB). However, to the best of our knowledge, the genetic effect of *CDH13* on DR has not been reported, thus far.

Furthermore, our results suggest the interaction effect of genes and APN. Teng et al reported that *CDH13* genotypes with low APN levels were associated with a more favorable metabolic profile but with a higher risk profile with respect to the levels of inflammatory markers. They suggested that APN acted as a suppressor of the association between CDH13 variants and various metabolic phenotypes in the mediation analysis.^[21,36]^ In the present study, the APN level and GRS were 2 independent risk factors but we did not observed any statistically significant interaction between them in the model.

T-cadherin, which is encoded by *CDH13*, is a GPI-anchored protein that binds with the hexameric and HMW forms of APN. Previous studies have shown the effect of T-cadherin on angiogenesis and revascularization.^[16,19]^ T-cadherin has been implicated in the modulation of angiogenic activities in cultured endothelial cells.^[16]^ The increased expression and ligation of T-cadherin on the cell surface induced by oxidative stress, inflammation, or prolonged exposure to insulin results in chronic stimulation of the Akt cascade and leads to insulin sensitivity in endothelial cells, attenuation of insulin-dependent angiogenesis, vasorelaxation, and progression of endothelial dysfuntion.^[18]^ Moreover, the upregulation of T-cadherin is involved in VE-cadherin phosphorylation and results in the regulation of endothelial permeability, which is a striking feature of the early stage of DR.^[37]^ Also, research shows that the BiP complexation with T-cadherin could promote endothelial cell proliferation and migration and is related to the formation of abnormal vasculature in the retina under ER stress.^[38]^

We identified some limitations in the present study. First, this is a cross-sectional study and no healthy control. The relationship between the plasma APN level and DR could not imply the causation. A prospective cohort study is needed to assess the role of APN in DR. Second, the sample size of plasma APN level was small in the present study, which might provide limited statistical power for detecting the effect of APN level on the DR status. Third, we do not have the information of the use of medication by the cases and the association between plasma APN level and DR could be confounded. Multiple comorbidities are common in people with diabetes and could be treated medically. And the increasing circulating APN level linked with the treatment of PPARγ agonists (thiazolidinediones) or fibric acid derivatives^[39]^ (bezafibrates or fenofibrates)^[32]^ were shown.

In conclusion, the association between plasma ANP level and the variance of APN related genes on the DR status, individually and in combination, was observed in the present study. Further experimental studies will be needed to validate the findings of this study and to provide a more comprehensive understanding of the underlying mechanisms of APN and related receptors on different stages of DR.

## Acknowledgments

We thank the National Center for Genome Medicine of the National Core Facility Program for Biotechnology, Ministry of Science and Technology, for the technical/ bioinformatics support.

## Author contributions

**Conceptualization:** Wen-Ling Liao.

**Formal analysis:** Ya-Wen Chang, Chia-Ming Wu, Ai-Ru Hsieh.

**Funding acquisition:** Wen-Ling Liao, Fuu-Jen Tsai.

**Methodology:** Yung-Hsiang Chen, Yu-Chuen Huang, Yng-Tay Chen.

**Project administration:** Chia-Ming Wu.

**Resources:** Ching-Chu Chen, Hui-Ju Ling, Bo Ban.

**Supervision:** Wen-Ling Liao.

**Validation:** Wen-Ling Liao.

**Writing-original draf:** Wen-Ling Liao.

## Supplementary Material

Supplemental Digital Content
