# Supplementary material for: Effect of adiponectin level and genetic variation of its receptors on diabetic retinopathy: A case–control study
Source: Medicine (Baltimore). 2019 Mar 15;98(11):e14878. doi: 10.1097/MD.0000000000014878 (PMC6426570; doi:10.1097/MD.0000000000014878)
Supplement: Supplemental Digital Content [file medi-98-e14878-s001.docx]

**
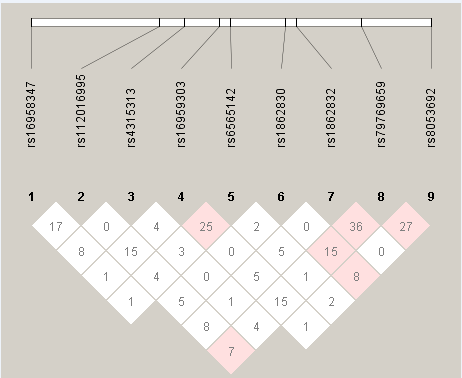
**

**Figure S1.** The pair-wise linkage disequilibrium (LD) between the 9 SNPs on CDH13 loci.


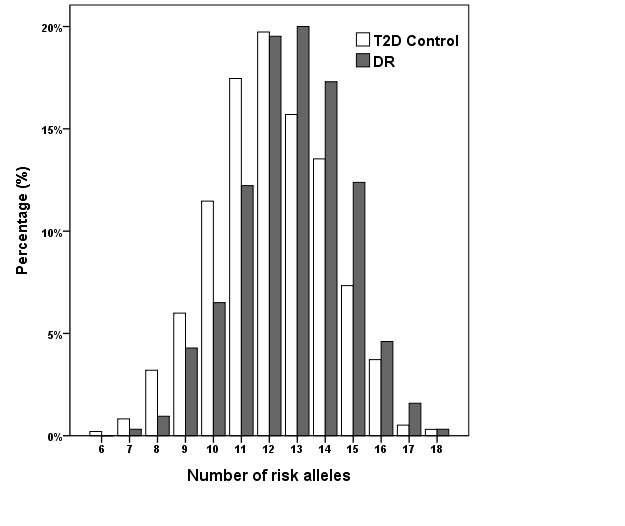


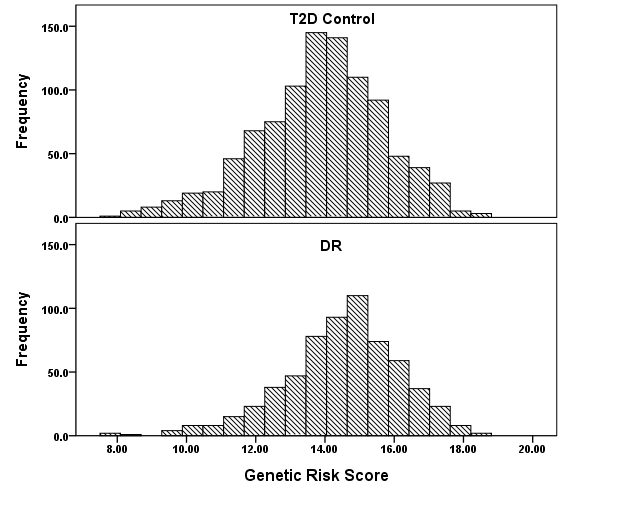


**Figure S2.** Distribution of the number of (A) risk alleles and (B) genetic risk score.
